# Role and Therapeutic Potential of Melatonin in the Central Nervous System and Cancers

**DOI:** 10.3390/cancers12061567

**Published:** 2020-06-13

**Authors:** Sangiliyandi Gurunathan, Min-Hee Kang, Jin-Hoi Kim

**Affiliations:** Department of Stem Cell and Regenerative Biotechnology, Konkuk University, Seoul 05029, Korea; pocachippo@gmail.com

**Keywords:** MLT, central nervous system, synthesis, brain damage, neuroprotection, antioxidative, anti-inflammatory, anticancer

## Abstract

Melatonin (MLT) is a powerful chronobiotic hormone that controls a multitude of circadian rhythms at several levels and, in recent times, has garnered considerable attention both from academia and industry. In several studies, MLT has been discussed as a potent neuroprotectant, anti-apoptotic, anti-inflammatory, and antioxidative agent with no serious undesired side effects. These characteristics raise hopes that it could be used in humans for central nervous system (CNS)-related disorders. MLT is mainly secreted in the mammalian pineal gland during the dark phase, and it is associated with circadian rhythms. However, the production of MLT is not only restricted to the pineal gland; it also occurs in the retina, Harderian glands, gut, ovary, testes, bone marrow, and lens. Although most studies are limited to investigating the role of MLT in the CNS and related disorders, we explored a considerable amount of the existing literature. The objectives of this comprehensive review were to evaluate the impact of MLT on the CNS from the published literature, specifically to address the biological functions and potential mechanism of action of MLT in the CNS. We document the effectiveness of MLT in various animal models of brain injury and its curative effects in humans. Furthermore, this review discusses the synthesis, biology, function, and role of MLT in brain damage, and as a neuroprotective, antioxidative, anti-inflammatory, and anticancer agent through a collection of experimental evidence. Finally, it focuses on the effect of MLT on several neurological diseases, particularly CNS-related injuries.

## 1. Introduction

The brain and spinal cord are both critical constitutes of the central nervous system (CNS). Injury or disease can lead to the degeneration of the CNS, including loss of homeostasis [1]. CNS injuries cause illness and death, including traumatic brain injury (TBI) and spinal cord injury (SCI), and such injuries eventually lead to lose of body functions [1,2,3,4]. CNS injuries ultimately cause blood–brain barrier (BBB) and blood spinal cord barrier disruption, mitochondrial dysfunction, neurotransmitter accumulation, and apoptosis. These defects follow the initial primary mechanical trauma [5,6]. The occurrence of CNS cancers has been increased between 1990 and 2016, and it affects both children and adults; CNS cancers cause illness and death of brain cancer worldwide [7]. The highest occurrence of CNS cancer was observed in East Asia. Most brain tumors are not linked with any known risk factors; however, one of the most significant risk factors for CNS cancer is exposure to radiation. Mostly, children are vulnerable to brain cancer during brain radiation treatment, as part of their treatment for leukemia. People with weakened immune systems are more prone to developing lymphomas of the brain or spinal cord. Besides these factors, other risk factors include industrial exposures, aspirin use, cell phone radiation, low-frequency magnetic fields, hormonal factors and hormonal imbalance, use of enormous pesticides in agriculture, and dietary factors [8,9]. 

Melatonin (MLT) is primarily secreted by the pineal gland from the essential amino acid tryptophan. Tryptophan is involved in both hydroxylation and decarboxylation processes for the synthesis of MLT. The synthesis of MLT is mainly influenced by the dark and light cycle. MLT is also produced by other organs, including the liver, stomach, bone marrow cells, lymphocytes, muscle, spleen, thymus, heart, intestine, and epithelial cells [10]. Recent studies have investigated its unique role in the CNS as a free-radical scavenger, neuroprotective, anti-inflammatory, and anti-apoptotic agent. MLT is produced in several parts of the mammalian body and regulates the chronobiotic actions of the circadian pacemaker and peripheral organs. Additionally, the function of MLT is not only restricted to circulation but also extends to direct effects in the CNS. MLT is a highly resourceful, multifaceted molecule and a pleiotropic regulator that orchestrates countless physiological functions [11,12,13,14]. MLT regulates various functions through MT1 and MT2, G protein-coupled MLT receptors, which are widely distributed within the brain [15,16,17]. MLT plays a major role in a variety of mechanisms, including the elimination of reactive oxygen species (ROS) and the reduction of the formation of ROS and reactive nitrogen species (RNS) [11,18,19]. MLT protects mitochondria by preventing excessive electron leakage and damage to components of the electron transport chain [20,21,22,23,24,25,26]. MLT controls high-grade inflammation, such as overactivation of innate immunity, loss of circadian control of mitochondria, enhanced free-radical formation, inflammasome activation, and overproduction of pro-inflammatory cytokines (such as IL-1β) due to alteration in the circadian oscillator function [27]. Although MLT exhibits various beneficial activities in the body, a lack of secretion or production of MLT or MLT receptor expression, and decreases in MLT levels, lead to numerous dysfunctions and diseases [28]. This review aims at providing a detailed overview of the current state of knowledge related to the role of MLT in the CNS and CNS related cancers and also at summarizing the protective role of MLT against various CNS related diseases.

## 2. Synthesis, Biology, and Functions of MLT

MLT was first isolated in 1958, by Lerner, from the extract of bovine pineal tissue [29,30]. MLT is an ancient molecule and phylogenetically old molecule with a life span of approximately 2.5–3.0 billion years. Initially, MLT function was described as a free-radical scavenger. MLT presumably evolved in bacteria, and structures were conserved in all organisms. The extensive distribution of MLT in various biological systems indicates that the chemical is a prehistoric molecule that has been conserved throughout the evolution of all organisms [31] 

The biological functions of MLT vary from species to species, depending on necessity. The hormonal functions of MLT regulate various tasks, such as reproductive activities, facilitation of sleep physiology, immunomodulation, promotion of stem cell proliferation, anti-inflammation, and modulation of aging [32]. MLT can modulate various physiologic processes, including mood regulation, the circadian clock in our body, anxiety, appetite, sleep, cardiac functions, and immunological responses. Moreover, MLT has been widely available in various foods, such as fungi, plants products, eggs, and fish. Particularly within plant foods, fruits and nuts have the highest content of MLT. Rich sources of MLT are essential for normal biological functions and the exhibition of various functions, including antioxidant, antidiabetic, anti-inflammatory, anti-obese, immunity booster, neuroprotective, and cardiovascular protective, anticancer, and anti-aging activities.

**Figure 1:**
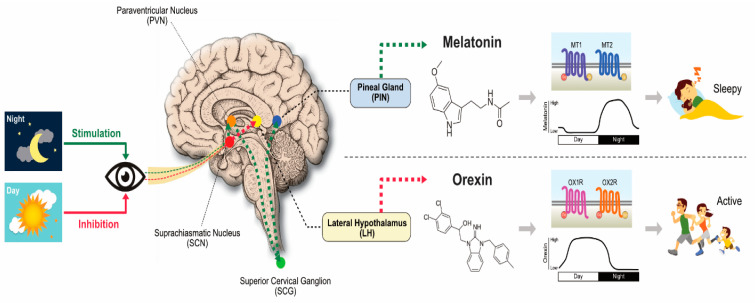
Melatonin (MLT) synthesis and mechanism of action in the biological system. The concept was adapted and modified from Reference [43].

MLT regulates various functions through the activation of G-protein-coupled receptors, such as MT1, MT2, and MT3. In mammals, MLT receptors are found in the brain and some peripheral organs [11,33,34]. MLT, which plays a critical role as a neuroprotectant and antioxidant in various physiological conditions, is primarily synthesized by using tryptophan as a precursor. The conversion of tryptophan into MLT goes through several intermediate steps, such as hydroxylation and decarboxylation, after which it is converted into serotonin. Serotonin is then acetylated by N-acetyltransferase to form N-acetyl serotonin (NAS). Finally, NAS is converted to N-acetyl-5-methoxytryptamine (MLT) by enzyme hydroxyindole-O-methyltransferase/acetylserotonin methyltransferase [35,36]. The synthesis of MLT is mainly influenced by the dark and light cycle (Figure 1)**.** MLT is mainly produced by the pineal gland and also other organs involved to produce MLT such as gastrointestinal tract, retina, tongue, skin, blood platelets, and bone marrow cells [37]. The majority of animal studies suggests that the duration of MLT synthesis is mostly influenced by the duration of the night, and the level of the hormone is high in dark and slowly decreases in the daytime. Pineal substances play a critical role in brain tumor growth. Many cells can produce MLT and NAS, including astrocytes [38], macrophages [39], fibroblasts, and skin cells [40]. The synthesis of MLT in astrocytes is mainly governed by the availability of serotonin and apolipoprotein. MLT is produced by extra pineal tissues, pineal glands, and other organs, such as Harderian gland, retina, bone marrow, gut, platelets, glial cells, astrocytes, pancreas, kidneys, and lymphocytes. Pinealocytes are the main source of MLT in blood and CNS, and MLT synthesis is well documented in animals and plants [41,42]. The rate of synthesis of MLT merely depends on the circadian and seasonal action of tryptophan, serotonin, arylalkylamine N-acetyltransferase, and tryptophan hydroxylase [30]. MLT serves as an immune enhancer, anti-inflammatory agent [43], free-radical scavenger [44], human mood- and behavior-modulating agent [45], and anticancer agent [46,47]. It is also involved in the oral cavity [48], traumatic CNS injury [2], an anti-angiogenic molecule in breast tumors [49], and sleep regulation [50]. MLT activity is mainly regulated and governed by MT1 and MT2, which are widely distributed in CNS and primarily found in suprachiasmatic nuclei in the hypothalamus of the mammalian cells.

The molecular activity of MLT was regulated by two high-affinity G protein–coupled receptors, termed MT1 and MT2, to exert beneficial actions in sleep and circadian abnormality, mood disorders, learning and memory, neuroprotection, drug abuse, and cancer. The clear understanding of the role of MLT receptors involved in various functions in the central nervous system led to the discovery of a novel class of MLT agonists for treating various type of diseases, such as insomnia, circadian rhythms, mood disorders, and cancer. MLT activates MLT receptors MT1 and MT2 in the CNS, to signal photoperiodic information and regulate physiological functions. Exogenous MLT modulates processes and responses in the central nervous system via activation of the MT1 and/or MT2. Further MLT activation of MT1 receptors decreases breast and prostate cancer cell growth [51]. MLT regulates various signaling molecules such as forskolin-stimulated cAMP, protein kinase A signaling, and CREB phosphorylation. The MT1 receptor also increases phosphorylation of mitogen-activated protein kinase 1/2 and extracellular signal–regulated kinase 1/2, as well as increasing potassium conductance [52]. MLT interacts with its surface receptors to activate intracellular signaling cascades, and it leads to the activation of transcription factors that cause changes to the DNA in a manner that enhances apoptosis of cancer/pre-cancer cells and reduces angiogenesis, which is necessary for tumor growth and metastasis. On the other hand, MLT could change the DNA to activate the upregulation of antioxidant defenses, downregulation of pro-oxidants, and modifications to immune responses that alter the microenvironment of cancer cells in a manner that reduces cancer progression and metastasis [53]. The most important activities of MLT, such as antioxidant and anti-inflammatory activities, play a critical oncostatic role by activating the MLT receptors. The activation of MLT receptors induces the activation of a wide range of transcription factors and signaling pathways. For example, activation of MT1 receptors by MLT inhibits stress-mediated cytochrome c release, which plays a crucial role in the prevention of neurodegeneration associated with mitochondrial cytochrome c release and downstream caspase activation [54]. Activation of MT3 receptors by MLT selectively causes cytotoxicity and promotes apoptosis in tumor cells [55].

## 3. Role of MLT in Brain and Spinal Cord Injuries

In the USA alone, 2.5 million people are afflicted with TBI, which is a devastating neurological deficit. As a result of TBI, various pathological and physiological damage was observed, such as contusion, vascular injury, axon shearing, immediate and irreversible disruption of neuronal cell bodies, and most symptomatic occurrences such as respiratory depression, apnea seizures, ischemia, hypoxia, parenchymal inflammation, and oxidative damage to lipids and elevated level of nitric oxide (NO) production [2]. The secondary injury is a result of the primary injury that constitutes a complex cascade of metabolic, physiologic, and biochemical factors that cause progressive tissue damage [6]. The primary and secondary mechanisms include complex consequences of the activation of pro-inflammatory cytokines, cerebral edema, upregulation of NF-κβ, disruption of BBB, and oxidative stress. SCI includes primary and secondary injury cascades that are a result of the generation of free radicals and oxidative or nitro-oxidative damage [56]. CNS injuries, including TBI and SCI, constitute a major cause of morbidity and mortality [2,3,4] and cause loss of body functions. As a result of TBI and SCI, several impairments occur in the CNS, including mitochondrial dysfunction, neurotransmitter accumulation, BBB and blood spinal cord barrier disruption, apoptosis, excitotoxic damage, and the initiation of inflammatory and immune processes [5,6].

As a result of secondary injury, excessive levels of ROS and RNS are generated, and this causes damage to macromolecules and the cell membrane and impairments in ionic homeostasis. The ionic pump is highly sensitive to ROS and LPO. Consequently, it increases the intracellular concentration of Ca^2+^, which is the primary cause of excitotoxicity, and it also causes overwhelming effects, including the production of free radicals. These metabolic alterations induce overactivation of phospholipases, calpains, protein kinases, and endonucleases [57,58].

MLT, a naturally occurring hormone, possesses various salient features, including low toxicity [59], the ability to cross the blood–brain barrier [60], and receptors that bind MLT in the CNS [61]. Preclinical studies demonstrated that MLT reduces injuries, symptoms, and functional aspects of various CNS disorders. MLT plays a critical role in brain damage; the brain uses a large amount of the total oxygen (O_2_) inhaled, even though the organ accounts for only 2% of the body weight. Generally, the antioxidative defense system in the brain is fragile and not equipped to overcome oxidative stress. MLT can cross the BBB. In contrast, typical free-radical scavengers such as vitamin C and E are not able to enter the brain, and these do not protect against acute oxidative/nitrosative stress in the brain [62]. Neural structures of the brain can uptake MLT rapidly, and the CNS can utilize high concentrations of MLT to protect against oxidative-stress-induced damage [63,64]. Several CNS disorder model studies including Parkinsonism, Alzheimer’s and Huntington’s disease, amyotrophic lateral sclerosis, neural trauma, and ischemia/reperfusion (I/R) injury demonstrated that MLT can protect the brain from various toxicities, such as chemical and neurotoxicity [43,65,66,67,68]. MLT rescues free-radical-induced brain damage I/R injury [66]. MLT protects not only the CNS but also against damage induced by free radicals in other organs. MLT protects against neural morphological loss and functional destruction and also provides a cerebral-protective effect by inducing upregulation of anti-apoptotic factors such as Bcl-2 and reduction of the pro-apoptotic factor Bax in ischemic injury [69,70]. Mitochondria is the main organelle for energy source, and if impaired, it leads to various neurodegenerative diseases. Therefore, maintaining mitochondrial homeostasis is critical for the normal physiological function of the cells. MLT can inhibit the release of cytochrome c and the loss of membrane potential; maintenance of cellular bio-energetic homeostasis and its dissipation leads to the formation of mPTP in stroke. MLT act as an anti-inflammatory agent by regulating the levels of NO, pro-inflammatory cytokines, and various enzymes like cyclooxygenase-2 (COX-2) and inducible nitric oxide synthase (iNOS) in various neurodegenerative diseases [71]. Furthermore, MLT can dissolve blood clots as a protective agent with tissue plasminogen-activator. MLT protects against acute and chronic cerebral ischemia due to disruptions of BBB and edema formation. MLT is a good free-radical scavenger that is effectively used as an anti-permeability agent to regulate membrane permeability and edema formation, and it can preserve the neural morphology, function, and integrity of the brain [72].

**Figure 2:**
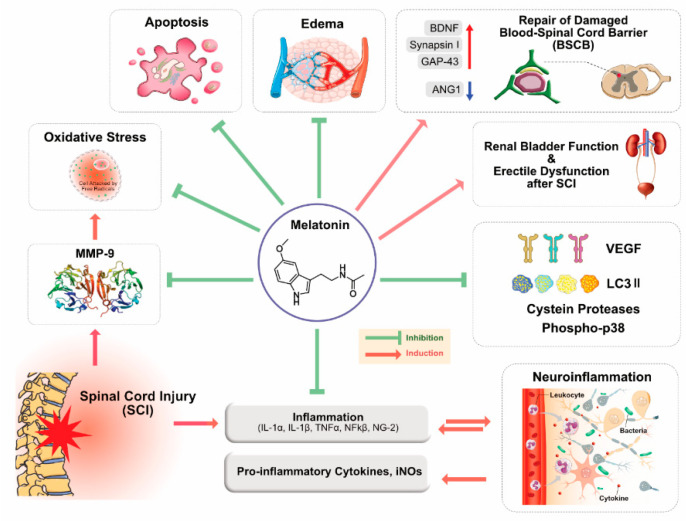
Potential mechanisms and beneficial effects of MLT in central nervous system (CNS)-related injuries. The concept was adapted and modified from Reference [75].

Primary injury of SCI is responsible for physical impacts such as alteration in cellular and molecular cascade of events, and it is also involved in free-radical-induced cell death, glutamate excitotoxicity, autoimmune response, vascular events, apoptosis, inflammation including release of pro-inflammatory cytokines IL-1β and TNF-α, and microglial activation [73]. The severity of SCI depends on a series of events including cellular, molecular, and biochemical reactions; tissue damage; calcium ion influx; oxidation of lipids; myelin degradation; and vascular changes inflammatory reaction and autoimmune response. Furthermore, SCI causes tissue damage as a result of activation of various proteases, including lipases, endonucleases, and metalloproteinases [74]. Excessive ROS production induces damage of various macromolecules, including unsaturated fats, lipids, proteins, and DNA, and it also induces secondary injury. Various beneficial effects of MLT are shown in Figure 2.

## 4. MLT: Antioxidant, Neuroprotectant, and Immunomodulatory Agent

Antioxidant, neuroprotectant, and immunomodulation are associated processes and linked with each other. Particularly, oxidative stress is the source of all dysfunctions. MLT’s natural molecules serve as antioxidant, neuroprotectant, and immunomodulator. As a result of mechanical injury, the CNS develops devastating injury effects, such as neuroinflammatory responses, including the influx of monocytes, activation of microglial cells, and activation of pro-inflammatory cytokines, such as IL-1α, IL-1β, IL-6, TNF-α, interferon-γ, and intracellular adhesion molecules. These events facilitate the induction of nitro-oxidative stress, inhibition of neurodegeneration, and also exacerbate cerebral damage and inflammation [76,77]. MLT has a potential role in deactivating the release of pro-inflammatory cytokines in TBI [43]. An in vivo rat model suggested that the administration of MLT decreases brain edema, BBB permeability, and ICP at 72 h after TBI, at both low and high doses [78]. A Sprague–Dawley rat model of TBI suggested that administration of MLT either alone (200 mg/kg) or in combination with uridine, reduced posttraumatic edema in various brain regions of the male rat [79]. MLT inhibits mRNA expression of NF-κβ and decreases AP-1 levels to half the basal level, both of which are involved in different pathological conditions, including TBI and ischemia [80,81]. As a result, TBI increases BBB permeability, thus leading to astrocytic dysfunction, inflammation-related mechanisms, and permeability to endogenous proteins, which may cause brain dysfunction [82,83]. MLT acts as a good neuroprotectant by balancing the level of pro-oxidants and antioxidants. For example, MLT regulates the level of glutathione (GSH) and lipid peroxide levels [84]. Daily administration of MLT increases superoxide dismutase (SOD) and glutathione peroxidase (GPX) activity, and it also decreases the malondialdehyde (MDA) level significantly within 72 h, thus protecting cerebral tissue against oxidative stress [78]. A study demonstrated that, as a result of SCI injury, pro-inflammatory cytokines such as interleukin 1 (IL-1α) and IL-1β are activated within a few hours in SCI [85]. Cytokines such as IL-6 and TNF-α play a critical role in pathogenesis of pro-inflammatory damage and immune and vascular responses, respectively [86]. Activation of the NF-κβ signaling pathway plays a crucial role in inflammation and is a pathophysiologic cause of spinal cord inflammatory response [86]. After 24 h, SCI injury causes an increased expression of the p65 subunit protein in the nuclear fractions of the spinal cord tissue [87]. MLT serve as a neuroprotectant by suppressing the expression of NF-κβ-regulated adhesion molecules and reducing the production of pro-inflammatory cytokines [88]; it also prevents neuronal death by improving the recovery function in injured neuronal cells [89]. Animal model studies demonstrated that MLT can potentially reduce the activation of inflammation and tissue injury [90,91]. An experimental study suggests that upregulation of NO, iNOS and increased mRNA expression was observed in damaged regions of the spinal cord in three days after injury, compared with the control group. An animal model study shows evidence that MLT could significantly reduce iNOS expression and NO production [92], whereas phosphorylation of stress-activated protein kinase ERK1/2 was significantly increased. Conversely, administration of 50 mg/kg MLT post spinal cord trauma significantly reduced activation of mitogen-activated protein kinases (MAPKs) p38, JNK, and ERK1/2 [93,94]. Ersahin et al. reported that MDA levels were significantly increased, and GSH levels were significantly reduced in the SCI group of animals, whereas administration of MLT (10 mg/kg) reduced the level of MDA and restored the decreased level of GSH in spinal cord tissue [89]. An increased level of endogenous colony stimulating factor decreased the level of oxidants in TBI; MLT was also an effective neuroprotective agent to treat Alzheimer-type dementia, which is a progressive fatal neurodegenerative disorder [95].

Oxidative stress is a condition of the imbalance between the levels of pro- and antioxidants. ROS are biphasic and contribute to beneficial effects such as cell proliferation, differentiation, and survival at the physiological level. However, their excessive production leads to adverse effects, including apoptotic and necrotic death of cells. ROS play an important role as secondary messengers in many intracellular signaling pathways, and also as mediators of oxidative damage and inflammation [96]. Excessive levels of ROS cause LPO, protein oxidation, and DNA damage and also increase the pro-inflammatory response [97,98]. Most of the neurological disorders include the oxidative-stress-mediated disease pathology, which can counteract the body’s antioxidant enzyme battery that scavenges the excessive ROS and maintains the cells under physiological conditions [99]. Oxidative stress is a major cause of the development of neurodegenerative diseases such as Alzheimer′s disease and Parkinsonism, as well as neurological conditions, including epileptic seizures, stroke, brain damage, and neurotrauma, due to the excessive generation of ROS and low antioxidative potential of the neuronal cells. Oxidative stress causes pathogenesis of CNS injuries such as stroke, TBI, and SCI. The CNS demands a high level of oxygen, and the unsaturated lipid contents of CNS can easily react with free radicals. When an excessive level of oxidative stress occurs in the CNS cells, lipids are more prevalent and easily susceptible to oxidative stress; oxidation of lipids releases 4-hydroxy-2-nonenal (4-HNE) and acrolein. This oxidative stress renders the CNS as a target tissue for the onset and pathogenesis of several CNS disorders [100,101]. Nuclear factor erythroid-2-related factor 2 (Nrf2) found in the CNS regulates the antioxidant response and is involved in a central role in the astrocyte-mediated protection of neurons from ROS [102]. MLT has a remarkable antioxidant property, and its production rate determines the susceptibility to a disease. MLT not only acts as free radical scavenger but also has antioxidative potential and stimulates the synthesis of antioxidative enzymes.

A study has revealed the antioxidative effects [103], while another showed the free-radical-scavenging ability of MLT [104]. Direct administration of MLT exhibited detoxification of OH and toxic oxygen-related peroxyl radical LOOP [105,106]. MLT administration is effective in various oxidative-stress-induced neurodegenerative diseases [107]. MLT exhibited a neuroprotective effect against sodium-arsenite-induced oxidative stress in the nigrostriatal dopaminergic system of the rat brain. Similarly, a study reported that MLT protects arsenite-induced peripheral neurotoxicity by using dorsal root ganglion explants [108,109]. Administration of sodium arsenite (5 mg/kg/day) increased the number of apoptotic germ cells and the levels of the biomarker of LPO and MDA, while reducing SOD, CAT, and GPx activities; sodium arsenite also induced testicular apoptosis and oxidative stress [110]. A major oxidative stress is caused by an abundance of polyunsaturated fatty acids (PUFA). To prove that MLT has a role in antioxidative property, an in vivo study demonstrated that MLT inhibited arsenite-mediated lipid breakdown in a concentration-dependent manner in rat brain [111]. Furthermore, MLT acted as an anti-genotoxic agent against human blood cells, protecting them from the exposure of the pro-oxidant actions of arsenite [112]. All of these studies clearly indicated that MLT could protect or prevent arsenite-induced oxidative stress, and MLT seems to be a superior therapeutic tool to reduce oxidative stress caused by arsenite in the CNS. MLT could neutralize LOOP effectively and is more effective than water-soluble vitamin E/Trolox in neutralizing LOOP [106]. MLT is capable of decreasing oxidative damage to membrane lipids [113], protein [114], and DNA [115]. MLT can increase the activities of the brain and liver mitochondrial respiratory complexes I and IV and also prevent the reduction in the activity of complexes I and IV due to mitochondrial damage and induced oxidative stress [116]. Several studies confirmed that MLT could intervene in various pathological processes associated with I/R injury by a free-radical scavenger and promote antioxidant enzymes [117,118]. MLT has dual functions on mitochondria as free-radical scavenger, and it also promote the activity of the antioxidant enzymes, including SOD, GPx, GRd, and catalase. Another function of MLT is to maintain GSH level in mitochondria and to enhance the production of ATP by enhancing the expression of complexes I and IV of the electron transport chain under normal conditions and restores their activities in some pathological situations; MLT also acts as an electron donor to certain proteins [117,119]. Hence, MLT is an excellent antioxidative molecule and also a promising agent for neuronal therapeutics.

**Figure 3:**
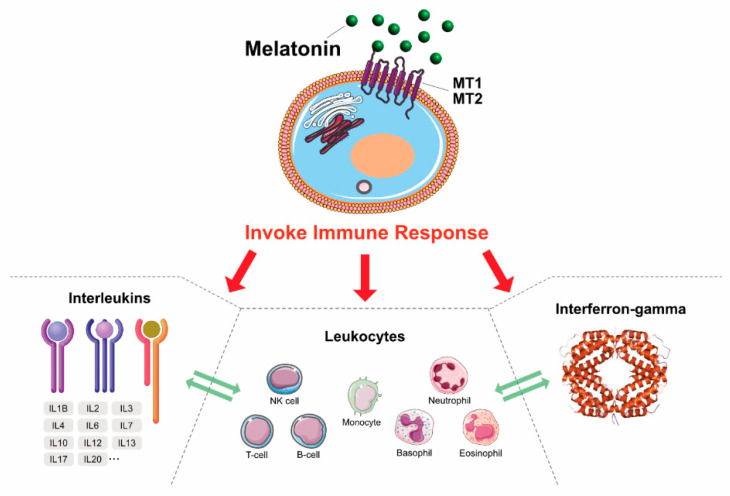
The immunomodulatory response of MLT in various immune cells.

MLT is a double-edged sword, which has both pro- and anti-inflammatory actions. Anti-inflammatory actions of MLT can be measured by inhibition of the activation of inflammatory cells via reduced myeloperoxidase activity. A study demonstrated that MLT protected sepsis-induced functional and biochemical changes in rat ileum and urinary bladder [120]. NO, COX-2, and myeloperoxidase (MPO) are important inflammatory mediators for cerebral I/R. A middle cerebral artery occlusion stroke model demonstrated that the administration of MLT significantly reduced the infarct volume in rats. Furthermore, pretreatment with MLT at 5 mg/kg provided neuroprotection against I/R injury, partly via inhibition of the consequential inflammation [121]. The mouse model consisted of control, inflammation (IA), and MLT-treated (IAM) groups. A study suggests that the IA group mice had a significantly elevated concentration of lipid peroxides, whereas a reduction in antioxidant enzyme levels, and also dopamine, 5-hydroxytryptamine, and norepinephrine, was observed. Interestingly, MLT treatment effectively reversed these abovementioned changes, normalized the lipid peroxide and antioxidant enzyme levels, and also recovered the brain cells from inflammation. This study concluded that the administration of MLT protects against inflammation associated with amyloid-beta vaccination [122]. MLT suppresses NLRP3 activation in radiation-induced damage of oral mucositis [123], small intestine toxicity [124], and subarachnoid hemorrhage [125]. NO mostly contributes to inflammation in the CNS. The anti-inflammatory actions of MLT depend on its inhibition of the expression of iNOS; MLT acts efficiently as an NO scavenger and forms a stable product that does not easily re-donate NO [126]. MLT exerts its anti-inflammatory activity by preventing the activation of the pro-inflammatory enzymes COX-2 and iNOS in glioma cells without simultaneous inhibition of the COX-1 enzyme [127] and reduces the expression of both components of the NLRP3 inflammasome and the levels of pro-inflammatory cytokines [125,128]. An in vitro model study using human dopaminergic neuroblastoma SH-SY5Y cells suggests that MLT could inhibit methamphetamine (METH)-induced iNOS expression, the levels of TNF-α mRNA, and phosphorylated nuclear factor-κB (NF-κB); MLT also downregulated Nrf2 [129]. A mouse model demonstrated that intraperitoneal injection of MLT (10 mg/kg) in SCI showed that long-term MLT treatment attenuated the inflammatory response, decreased IL-1β and neuron/glial antigen 2 levels, and can also prevent the secondary inflammatory response and tissue damage [130]. Long-term treatment of MLT provides protective effects by preserving cell and nerve structures. MLT alleviates post-traumatic injury associated with SCI by binding the PPAR-α receptors [131]. MLT potentially reduces histological damage, nuclear factor of kappa light polypeptide gene enhancer in B-cells inhibitor-alpha degradation, nuclear factor κB activation, polymorphonuclear leukocyte infiltration, and MAPK activation in the injured spinal cord [91]. TNF-α is a critical factor for inflammation in the acute phase of SCI. The administration of MLT in radiation-induced rat potentially reduces the expression of TNF-α expression after radiation-induced SCI [132]. MLT acts as both a pro-inflammatory and anti-inflammatory agent, depending on the requirement of the cells, and it activates various immunological response cells (Figure 3).

## 5. MLT as an Anticancer Agent

A study reported that concomitant administration of MLT in patients with glioblastoma treated with radical or adjuvant radiotherapy (RT) with MLT showed significant survival as compared to RT alone. Therefore, a radio neuroendocrine approach with RT plus MLT may prolong the survival time and improve the quality of life of patients affected by glioblastoma [133]. A clinical study demonstrated that the concomitant administration of aloe with MLT enhanced the therapeutic results of MLT in patients with advanced solid tumors. Additionally, patients with lung cancer, gastrointestinal tract tumors, breast cancer, or brain glioblastoma treated with MLT alone (20 mg/day orally) or MLT plus *A. vera* tincture (1 mL twice/day) achieved significant positive responses [134]. Exogenous supplementation of MLT (3 mg) for two weeks reversed the sleep disorder in a child with a germ cell tumor involving the pineal region, which is primarily responsible for the suppression of MLT secretion associated with severe insomnia [135]. MLT potentially inhibits oxidative stress and neurotoxic effects induced by the amyloid beta protein (A beta) by preventing the death of cultured neuroblastoma cells [136]. Oral MLT supplementation for the child with the diagnosis of a pineal tumor, severe reduction of secretion of MLT, and lack of sleep significantly improved her sleep, without any adverse effects. MLT replacement therapy is beneficial for the patients with deficient MLT synthesis and sleep disorder [137]. A study demonstrated that, in female CBA mice supplemented with MLT (20 mg/L) for five consecutive days every month, the consumption of MLT did not significantly influence food consumption. However, it did increase the bodyweight of older mice and decreased locomotor activity and body temperature.

Further analysis revealed that MLT decreases the level of free radicals in the serum, brain, and liver and eventually increases the life span [138]. Granzotto et al. reported that the attenuated effect of MLT on doxorubicin (DOX) induced toxicity in a variety of human cancer cell lines, including human normal mammary epithelium HBL-100, mammary adenocarcinoma MCF-7, colon carcinoma LoVo, mouse P388 leukemia cell lines, and tumor cell sublines pleiotropically resistant to anthracyclines [139]. The results depicted that MLT causes an inhibition of the growth of the human cell lines in the concentration range 10–2000 pg/mL, but not in a dose-dependent manner. Conversely, 200–1000 pg/mL MLT causes a significant and dose-dependent partial sensitization to DOX in resistant P388 mouse leukemia (P388/ADR) both in vitro and in vivo. The in vitro studies proved that MLT induces apoptosis of rat pituitary prolactinoma cells. A rat model with 17-beta-estradiol (E2)-induced pituitary prolactin-secreting tumor was inhibited with MLT, and also daylight illumination shows potential impact by inhibition of proliferation and increased cell apoptosis through induced mRNA expression of Bax and cytochrome c protein expression in prolactinoma cell, whereas anti-apoptotic proteins were significantly reduced [140].

Animal studies suggest that administration of MLT with dose of 15 mg/kg body weight of MLT to rats injected with C6 glioma cells reduces tumor growth. Further evidence from in vitro studies suggested that MLT reduced cell growth of C6 glioma cells by inhibiting cell progression from G (1) to S phase of the cell cycle; MLT also inhibited cell growth [141]. In addition, another study demonstrated that MLT inhibits cell viability and induces apoptosis through the accumulation of cells in the G2/M cell cycle phase and increasing leakage of lactate dehydrogenase and caspase-3 activation [142]. Combination of cisplatin (CDDP) and etoposide or CDDP plus gemcitabine with MLT showed a significant impact in non-small-cell lung cancer (NSCLC) or gastrointestinal tumors patients. Similarly, the combination of oxaliplatin plus 5-fluorouracil (5-FU) and MLT exhibited significant tumor reduction in colorectal cancer patients. These findings suggest that anticancer therapeutic properties of MLT enhance the efficacy of the standard anticancer chemotherapies [143].

**Figure 4:**
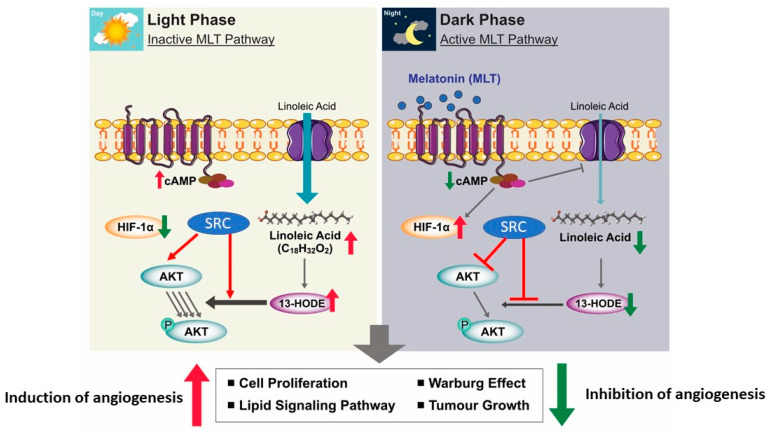
Differential anticancer effect of MLT in the presence and absence of light. The concept was adapted and modified from Reference [148].

Patients with untreatable metastatic solid tumors treated with exogenous MLT alone or MLT plus subcutaneous low-dose interleukin-2 exhibited tumor progression on NSCLC or gastrointestinal tract tumors and increased survival time. The association of lL-2 with MLT increases the percentage of tumor regressions [144]. A combination of MLT and vincristine and ifosfamide showed a synergistic antitumor effect on SK-N-MC human Ewing sarcoma cancer cells, through extrinsic apoptosis [145]. Combinations of MLT and temozolomide showed a significant synergistic toxic effect on BTSCs and A172 malignant glioma cells by downregulation of the ABC transporter ABCG2/BCRP [146]. A combination of MLT and chemotherapeutic drugs exhibited a synergistic toxic effect on A172 malignant glioma cells and brain tumor stem cells via downregulation of the expression and function of adenosine triphosphate (ATP)-binding cassette transporter ABCG2/BCRP [146]. MLT potentially inhibits tumor development, progression, therapeutic resistance, and tumor relapse by MLT alone and in combination with a chemotherapeutic drug. MLT has the ability to suppress cAMP formation; it inhibits the uptake of LA and its metabolism to 13-Hydroxyoctadecadienoic acid (13-HODE) by a MEL-receptor-mediated mechanism found in rat hepatomas and human breast cancer xenografts [147]. MLT has the potential ability to regulate cell proliferation, lipid signaling, Warburg effect, and tumor growth depending on dark and light phase (Figure 4).

Furthermore, MLT treatment reduces glioblastoma-initiating cell proliferation and induces a decrease in self-renewal and clonogenic ability, accompanied by a reduction in the expression of stem cell markers. Hence, MLT can be used as a potential therapeutic agent for malignant glioblastoma [149]. MLT competently inhibits methamphetamine (METH)-induced neurotoxicity in rat glioma cell line (C6 cells) through the reduction of oxidative stress, nitro-oxidative stress, and inflammation by suppression of NF-κB and translocation of the NF-κB (p65) subunit into the nucleus. Furthermore, MLT pretreatment promoted Nrf2 activation, reversed the METH-induced NF-κB response, and increased SOD activity. This study suggests that MLT reduces the pro-inflammatory responses induced by inhibiting NF-κB activation and inducing Nrf2-mediated HO-1, NQO-1, and γ-GCLC expression in C6 cells [150].

MLT also protects cadmium-induced neurotoxicity of mitochondrial damage in cortical neurons and brain tissues. Exposure of cortical neurons to 10 µM of cadmium causes an imbalance in mitochondrial dynamics by altering the structure and morphology of mitochondria, fragmentation of mitochondria, production of excessive ROS, decreased ATP content, and loss of mitochondrial membrane potential (MΨm). Interestingly, 1 mM MLT pretreatment efficiently attenuated cadmium-induced mitochondrial fragmentation, which improved the turnover of mitochondrial function in cortical neurons. Similarly, MLT protects the brain tissues of rats exposed to 1 mg/kg CdCl_2_ for seven days. As a result, MLT ameliorated excessive mitochondrial fragmentation and mitochondrial damage in rats. Thus, the results suggest that MLT prevents abnormal mitochondrial dynamics caused by cadmium [151]. MLT significantly suppresses the release of the cytokine, metastasis, and invasion under hypoxic stress in glioma cells [152]. MLT inhibits conditioned-medium-induced angiogenesis in endothelial cells [153].

Recent studies have highlighted combination therapy as an attractive approach to reduce severe toxicity in normal cells and drug resistance in cancers by chemotherapy. Lee et al. investigated the underlying antitumor mechanism of MLT and its potency as a sensitizer of paclitaxel in X02 cancer stem cells [154]. MLT suppressed sphere formation and induced G2/M arrest in X02 cells expressing nestin, CD133, CXCR4, and SOX-2. MLT reduced the expression of CDK2, CDK4, cyclin D1, cyclin E, and c-Myc and upregulated cyclin B1 in X02 cells. Finally, the study concluded that MLT synergistically promotes the sensitivity of paclitaxel, to increase cytotoxicity, sub-G1 accumulation, and apoptotic body morphology, as well as decrease the expression of Nestin and c-Myc in X02 cells. These results indicate the potency of MLT as a paclitaxel sensitizer, since long-term RT is one of the treatments for cancer. However, a recent study suggests the combination of MLT with RT as a treatment for colorectal cancer. The study revealed that MLT significantly inhibited human colorectal carcinoma cell line HCT 116 cellular proliferation, colony formation rate, and cell migration counts following IR.

MLT potentiates the radio sensitivity of colorectal cancer cells to induce apoptosis by cell-cycle-arrest downregulation of proteins and activation of the caspase. The combined treatment of MLT and IR exhibited significant suppression of tumor cell growth compared with either MLT or IR alone. Similarly, the combination of vorinostat and MLT induces apoptosis by promoting the oligomerization of transcription factor EB proteins, inhibition of CSC properties, and proliferation of GSCs, and it also induced the expression of cleaved PARP and p-γH2AX in glioblastoma cells [155]. The anticonvulsant effect of MLT in pentylenetetrazole (PTZ)-induced seizures in mice was investigated by acute intraperitoneal administration of MLT (40 and 80 mg/kg). MLT increased the latency of clonic seizures and reduced its frequency in mice receiving an intraperitoneal injection of PTZ. Using glibenclamide as a K_ATP_ channels blocker and diazoxide and cromakalim as K_ATP_ channels openers increased the anti-seizure effect of MLT in the PTZ model of seizures. These findings suggest that the anti-seizure effect of MLT was achieved by increasing the opening of K_ATP_ channels. The targets of ATP-sensitive potassium (K_ATP_) channels are important to determine, as they are considered to be essential in the modulation of seizures [156].

## 6. CNS Cancers

CNS is composed of the brain, spinal cord, and retina, and CNS disease is complex and has limited treatment options for patients with CNS injuries or diseases. Glioma tumor growth is the common malignancy with high invasiveness in the central nervous system. Interestingly, MLT (1 mM) was able to inhibit glioma cell growth in both human tissue specimens and two different glioma cell lines [157]. Metastasis of malignancies to the brain represents a common neurological complication in cancer patients [158,159]. Currently, 10% of all cancer patients experience involvement of the central nervous system (CNS) [160]. Though 40% of patients with metastatic cancers are affected by brain metastases [161], surgery and radiotherapy are commonly used treatment options for brain metastases [162]. Recently, CNS cancers were treated with various therapeutic approaches, such as chemotherapy, gene therapy, immunotherapy, phototherapy, and thermotherapy [163]. Chemotherapy was the most common treatment for brain cancer. Single chemotherapy facilitated the drug resistance of the tumor after a certain period, which greatly hindered the successful treatment of brain cancer. A combination of anticancer drugs improved the brain cancer therapy [164]. Radiotherapy was one of the clinical treatments for CNS cancers. Since the combination of radiotherapy with chemotherapy is standard treatment strategy to inhibit brain cancer [165], the radiotherapy concomitant with free temozolomide or carmustine entered into clinical trials. Phototherapy was composed of the photothermal therapy (PTT) and the photodynamic therapy (PDT), which was a promising non-invasive strategy for cancer treatments. Phototherapy treatment induces cancer-cell death through generation of reactive oxygen species [166]. Immunotherapy seems to be an important therapy for the treatment of various types of cancers, including CNS cancers. It could activate the body’s own immune systems and induce the specific immune responses with tumor antigens, to eliminate the tumor cells [167,168]. Immunotherapy facilitates the long-term reduction of cancer metastasis and recurrence [169]. The immunotherapeutic methods cover a variety of approaches, including the cancer vaccines, monoclonal antibodies, oncolytic virus, engineered T cells, and immunomodulation [169]. However, immunotherapy has several challenges, such as a lack of specific tumor antigens and limited immunogenicity of the cancer cells and the immunosuppressive environment of the tumors [170,171]. Besides these, hyperthermia therapy seems to be safe and effective complementary therapy for cancer treatments. The combination of magnetic and thermal therapy is more effective against brain cancer [172]. Similarly, the combination of photoimmunotherapy exhibited a better effect compared to either phototherapy or immunotherapy against brain cancer. The combination therapy uses the specific antibodies that could facilitate targeting the tumor cells without damaging the normal cells and also uses enhanced photosensitizer delivery into tumors and increases the light-activated cytotoxicity [173]. Recently, gene therapy was used as an adjuvant for the current glioblastoma treatments. The combination of gene therapy with immunotherapy shows better results of glioblastoma treatments [174,175].

Programmed death ligand 1 (PD-L1) is a major prognostic biomarker for immune therapy in many cancers, and it is widely expressed on various cell types, such as tumor cells, monocytes, macrophages, natural killer (NK) cells, dendritic cells (DCs), and activated T cells, as well as on immune-privileged sites such as the brain, cornea, and retina; it also plays a critical role in tumor progression and immunotherapy [176]. A recent study suggested that the activation of the PD-1/PD-L1 signaling pathway can arrest the T-cell cycle at the G1 phase rather than directly causing apoptosis [177]. For instance, exosomes obtained from MLT-treated hepatocellular carcinoma cells were able to downregulate the expression of PD-L1 and the secretion of cytokines (IL-6, IL-1β, IL-10, and TNF-α) in macrophages [178]. Inhibition of the PD-1/PD-L1 pathway by using antibodies shows a significant impact in advanced melanoma, including in its brain metastasis [179,180]. Clinical studies suggest that the overall effect of PD-1 antibody therapy in glioma is limited, particularly in the presence of MLT. However, a combination of immune therapy, including PD-1/PD-L1 antibodies, and molecular-targeted antitumor drugs may be a future direction of brain cancer therapy. Altogether, all of these therapies show a potential effect against CNS-related cancers.

There is a significant amount of evidence from human, animal, and cellular studies that shows that MLT could inhibit tumor growth and progression. MLT mitigates the pathogenesis of various types of cancer. Particularly, MLT is involved in preventing the initiation of the tumorigenic pathway and the ability of MLT to retard the progression of cancer [53,181]. Potential ability of antioxidant and immunomodulatory effects of MLT could modulate various gene expression following activation of various transcription factors in CNS cancers, which are likely to be an important mediating event in cancer prevention. MLT’s epigenetic activity is responsible for its antineoplastic and epigenetic effects, which are able to control CNS-related cancers. For example, MLT significantly inhibited the viability and self-renewal ability of glioblastoma stem-like cells (GSCs), accompanied by a decrease of stem cell markers [182]. The combination of MLT and a chemotherapeutic agent exhibited a potential synergistic toxic effect against A172 malignant glioma cells and brain tumor stem cells via downregulating the expression and function of adenosine triphosphate-binding cassette transporter ABCG2/BCRP [146]. MLT triggering various transcription factors and signaling pathways inhibits the proliferation and invasiveness of cancer progression. The MT1/MT2 receptor antagonist luzindole inhibits the expression of MMP-2 and MMP-9 genes, which regulate metalloproteinase activity in endometrial cancer [183]. The inhibitory effect of MLT on glioma cell lines is proportional to the expression level of MT1 in glioma [157]. Altogether, all of these studies indicate that MLT and its receptors play a significant role in CNS cancer prevention. Previously, several studies suggested that MLT inhibited the gene expression of MMP-9 in head and neck cancers, which is majorly responsible for metastasis [184,185]. MLT targeted the ERK/JNK pathways to reduce MMP-9 transcription and cancer cell invasion through modulating histone acetylation and SP1 activation in head and neck cancer [184]. Single-nucleotide polymorphism (SNP) occurs as a variation in one nucleotide, which occurs at specific locations in the genome, SNPs in genes involved in cell cycle control ECM remodeling, DNA repair, folate metabolism, and that carcinogen metabolism may be associated with increased susceptibility to cancers [186]. MLT exhibits oncostatic properties in many cancer types mainly mediated by its membrane-bound receptors, such as MT1/MT2 A. Increased expressions of MT1/MT2 promoted the inhibitory actions of MLT on the growth of cancer cells, and the variations of MLT receptor genes are associated with susceptibility to many diseases, including head and neck cancers [187,188]. The combination of polymorphism in the MLT receptor gene and environmental parameters increases the risk for cancer and eventually leads to reduce the survival rate of cancer patients.

## 7. MLT: A Novel Therapeutic Agent for the Treatment of CNS Disorders and Cancers

MLT affects every system of the body. MLT regulates various functions associated with the circadian rhythm in the dark phase, such as blood pressure, sleep induction, induction of insulin resistance and glucose intolerance, regulation of body temperature, and blockade of cortisol secretion [189]. MLT plays a critical role in cardiovascular-related disorders, including hypertension, ischemia, and valvular heart diseases [189,190]. MLT does not only provide neuroprotection in the CNS but also acts as an antioxidant and promotes the synthesis of antioxidant enzymes [189,191]. The concentration of MLT is directly associated with neurologic disorders such as TBI [192], mood disorders [193], and delirium [194]. The use of MLT as adjuvant therapy is most warranted in patients with neurologic disorders [189]. For example, a reduced level of MLT causes various metabolic disorders, such as glucose intolerance and insulin resistance, sleep disorders, metabolic syndrome, hypertension, and increased diabetes and cancer risk. Conversely, overproduction of MLT also causes various disorders, including hypogonadotrophic hypogonadism, Rabson–Mendenhall syndrome, anorexia nervosa, spontaneous hypothermia hyperhidrosis, and polycystic ovarian syndrome. 

BBB damage leads to a variety of neurological diseases in older patients, including acute and chronic cerebral ischemia and Alzheimer’s disease [38]. The level of MLT was significantly lower in older people, and this lower level of MLT causes BBB damage by the degradation of tight junction proteins [195]. The supplementation of MLT protects BBB in neuronal cells. Hu et al. demonstrated that MLT-mediated TLR4/NF-κB-signaling pathway was an important signaling cascade involved in BBB integrity [196]. This study suggests that MLT seems to be a suitable candidate as a therapeutic agent for protecting the CNS neurological diseases characterized by a compromised BBB. The dysregulation of MLT signaling in the brain constitutes a causal risk factor to neurodegenerative diseases and mood disorders. MLT restores sepsis-related inflammatory response and neurological complications [197]. MLT has been shown to restore the mitochondrial production of ATP in septic mice [198]. For example, MLT improved cardiac mitochondria and the survival rate in rat septic heart injury [74] through the inhibition of iNOS and the preservation of neuronal nitric oxide synthase [199], and attenuated sepsis-induced cardiac dysfunction via a PI3K/Akt-dependent mechanism [200]. The decreased level of MLT causes BBB damage in older people, which leads to sepsis and neuroinflammation. MLT supplementation treatment significantly inhibits sepsis and neuroinflammation. MLT is effectively used to treat sleep disorders and phase shift of circadian rhythms, depressive disorders, and improve learning and memory [201,202,203]. Studies demonstrated that MLT protects against neurodegeneration, apoptosis, and I/R injury by the mechanism of free-radical-scavenging properties [204,205].

In clinical trials, the anticancer effect of MLT was reported as an adjuvant therapy with other chemotherapeutic drugs. Several clinical studies suggested that MLT could enhance the therapeutic efficacy and reduce the toxicities of other anticancer drugs, as shown by increased partial response, induced tumor regression, higher survival rate, and relieved symptoms of side effects [188]. Combination of low-dose subcutaneous interleukin-2 and MLT significantly increased the one-year survival rate of patients with metastatic colorectal cancer, compared with supportive care alone [206]. In a phase-II study including 14 patients with metastatic breast cancer, the patients were free from anxiety [207]. MLT potentiates the effect of chemotherapeutic agents and reduces the toxicity in patients with metastatic solid tumor [208]. Concomitant administration of MLT on metastatic NSCL cancer patients receiving cisplatin and etoposide exhibited tumor regression rate and increased survival rate [209]. Similarly, concomitant administration of MLT with irinotecan on metastatic colorectal cancer patients achieved a higher percent of disease-control on patients than irinotecan alone [210]. Meta-analysis data revealed that 21 clinical trials, which all dealt with solid tumors, revealed that MLT as an adjuvant cancer care with chemotherapy decreased one-year mortality and reduced chemotherapy-induced symptoms, such as asthenia, leucopenia, nausea, vomiting, and hypotension [211]. MLT exhibited positive effect in tumor therapeutic strategies, including improving tumor remission rate and overall survival rate, while reducing the incidence of chemotherapy side effects [212]. Collectively, in clinical trials, MLT showed the ability to enhance the therapeutic effect of various anticancer drugs. Meanwhile, MLT might help improve the sleep and quality of life of cancer patients. Hence, MLT seems to be an efficacious alternative therapeutic agent for the treatment of CNS disorders and cancers.

## 8. Conclusion and Future Perspectives

MLT is a versatile hormone that is primarily secreted by the pineal gland in the human brain and is known to regulate various physiological processes by photoperiodicity. MLT exhibits a wide range of psychophysiological functions through the activation of MLT receptors, such as MT1 and MT2. Several studies have demonstrated the beneficial effects of MLT in different types of cancers. Here, we documented the overall effect of MLT on various activities, including its synthesis, biology, and functions; the role of MLT in brain damage as a neuroprotectant and antioxidative; and its anti-inflammatory and anticancer properties. Furthermore, we discussed the underlying mechanisms involved in the beneficial effect of its treatment of CNS and CNS-related disorders. In particular, MLT differentially regulates anticancer effects in the presence and absence of light. Several preclinical studies suggest that MLT exhibits neuroprotective effects and promotes the restoration of neurologic function after SCI. Although several studies have documented the mechanisms that are involved in the regulation of various beneficial effects as described in animal models, additional studies are needed to increase the therapeutic efficacy of MLT with a precise drug for cancer therapy. This review further emphasizes that more studies are required to address the administration of MLT to achieve the desired effect and to avoid undesired side effects; dosage and formulation should be carefully tailored to each individual. Systematic research is needed to understand and establish the connection between MLT and specific aspects of the CNS. Therefore, further studies should be directed at clarifying the cellular and molecular mechanisms that underlie the therapeutic effects of MLT and providing evidence-based rationales for the development of clinical therapies that can be used in CNS and CNS-related-disorder patients. Additionally, the development of MLT-receptors-based drugs is essential for the treatment of sleep and circadian rhythm disorders, depression disorders, learning and memory, neuroprotection, and various types of cancers. The design and development of new drugs targeting the MT1 and MT2 MLT receptors is the next challenge for the field of MLT.
